# 

**Published:** 1918-06-01

**Authors:** 


### CONTENTS.

PAGE

**Devil's Work** ... ... ... ... ... ... ... 173

**The London and Counties Medical Protection Society, Limited** ... ... ... ... ... 174

**Hospital and Institutional News** ... ... ... 175

Sir Arthur Sloggett, K.C.B., K.C.M.G., K.C.V.O.—The New D.G.M.S. in France—The New Secretary at Hartshill—A Hospital Constitution Resting on Complete Democracy—Death of a Former Hospital Chairman—Secretaries and the Tribunals—For the Fourth Time of Asking—" A Free Hospital Service "—Women Hospital Secretaries—William Dudley's Charity—A Will and the War—The Married Assistant Medical Officer—Permits for Surgical Instruments—" Colis " for Captured Printers—Women and the Housing Problem—The First Public Woman Dentist—A Trophy of Captivity—This Week's Drug Market.

**Dr. Henry Hurd's Wonderful Book** ... ... 179

The Institutional Care of the Insane in the United States and Canada.

**Physical Culture in Education** ... ... ... 181

III. The Re-education of the Defective.

**The Public Spirit of Property Owners** ... 182

**The Institutional Library** ... ... ... ... 183

PAGE

**Why Working Nurses Should Articulate** ... 185

The Hon. Sir Arthur Stanley's Reply to " Ierne's " Open Letter.

**Nursing Affairs in Ireland** ... ... ... ... 186

A National Tribute Fund.

**The Matrons' and Sisters' Department** ... 187

XIV. The College Conferences and Subsequent Gains.

Round the Hospitals.

The Nurse's Charter of Liberty—The College Conference—Tribute Fund for Irish Nurses—South Africa's V.A.D.—Club-house for Glasgow Nurses—Australian Nurses' Earnings.

**The College of Nursing (Limited by Guarantee)** 191

Sheffield Whole-heartedly Forms a Local Centre.

**Cost of Military Patients in Civil Hospitals** ... 192

The Inquiry Upon Which the Present Grant was Based.

**Recent Official Publications** ... ... ... 193

**The College of Nursing (Limited by Guarantee)** 194

**Matrons' and Sisters' Appointments** ... .. 194

**Annual Meetings of Four Children's Hospitals** 194

**Annual Meetings** ... ... ... ... ... ... 194

**The Institutional Worker** ... ... (Suppl.) 1

